# A Human Lower Limb Mechanical Phantom for the Testing of Knee Exoskeletons

**DOI:** 10.1109/TNSRE.2023.3276424

**Published:** 2023-06-01

**Authors:** W. Sebastian Barrutia, James Bratt, Daniel P. Ferris

**Affiliations:** Human Neuromechanics Laboratory, J. Crayton Pruitt Family Department of Biomedical Engineering, Herbert Wertheim College of Engineering, University of Florida, Gainesville, FL 32608 USA

**Keywords:** Ballistic gel, knee exoskeleton, lower limb, mechanical phantom, soft tissue deformation, stiffness

## Abstract

The development of assistive lower-limb exoskeletons can be time-consuming. Testing prototype medical devices on vulnerable populations such as children also has safety concerns. Mechanical phantoms replicating the lower-limb kinematics provide an alternative for the fast validation and iteration of exoskeletons. However, most phantoms treat the limbs as rigid bodies and fail to capture soft tissue deformation at the human/exoskeleton interface. Human soft tissue can absorb and dissipate energy when compressed, leading to a mismatch between simulated and human exoskeleton testing outcomes. We have developed a methodology for quickly testing and validating the performance of knee exoskeletons using a mechanical phantom capable of emulating knee kinematics soft-tissue deformation of the lower-limb. Our phantom consisted of 3D-printed bones surrounded by ballistic gel. A motorized hexapod moved the knee to follow a walking trajectory. A custom inverse dynamics model estimated the knee assistance moment from marker and load cell data. We applied this methodology to quantify the effects of soft-tissue deformation on exoskeleton assistance by loading the phantom knee with a torsional spring exoskeleton interfacing and bypassing the ballistic gel. We found that including soft-tissue deformation led to a lower knee assistance moment and stiffness. Some but not all of this difference could be explained by the deflection of the exoskeleton relative to the knee angle, suggesting energy absorption within soft tissue. The direct measurements of exoskeleton assistance provide insight into increasing the assistive moment transmission efficacy. The phantom provided a relatively accurate framework for knee exoskeleton testing, aiding future exoskeleton design.

## I. INTRODUCTION

Exoeskeletons, wearable mechanical devices worn in parallel with the human body, have become more commonplace in recent years [1], [2]. Human performance augmentations exoskeletons can increase user endurance, strength, and/or functionality of human movement. With this goal in mind, engineers have developed robotic lower limb exoskeletons that can aid healthy human users by reducing the muscle activation and muscle work required to perform some tasks [3], [4], [5]. Such devices can help users carry heavier loads and walk longer distances without tiring as much. Other robotic exoskeletons replace lost function for individuals with neurological or musculoskeletal deficiencies, or train humans with these deficiencies via therapeutic interventions. References [6] and [7] These rehabilitation exoskeletons may aid individuals with neuromuscular disorders and injuries such as cerebral palsy, spina bifida, multiple sclerosis, poststroke hemiparesis, or spinal cord injury.

Developing a robotic exoskeleton is typically a long process with many challenges and obstacles to overcome. Construction of new exoskeletons involves an iterative design process, resulting in fabrication of a prototype followed by human experiments to assess its functionality and assistance, and then looping back to redesign and refabricate a new prototype. It can be time-consuming to recruit subjects and evaluate the functionality of every exoskeleton iteration, especially when there are potential safety considerations for the patient populations involved [8]. For example, rehabilitation exoskeletons designed for children have the challenge of testing on a vulnerable population and difficult recruiting of test subjects. Computational models can speed up some aspects of exoskeleton development but they often do not simulate all the interaction forces, mechanical efficacy, and reliability of the device [9], [10], [11].

An alternate approach is to use a mechanical simulator to evaluate exoskeleton assistance and reliability prior to human trials. Shamaei et al. developed a mechanical knee simulator to ensure their knee exoskeleton could withstand the dynamic loads expected in walking [12]. The simulator consisted of a four-bar linkage driven by a servomotor to emulate the sagittal knee kinematics of walking. Goo et al. constructed a dummy leg for preliminary validation of their hip and knee pediatric knee exoskeleton actuator [13]. The leg consisted of a double pendulum with similar physical properties to that of a child. Similarly, Bregman et al. developed a mechanical leg for the mechanical characterization of ankle foot orthoses [14]. More recently, Yoshida et al. developed a humanoid robot to test active and passive wearable assistive devices [15], [16].

While useful for preliminary exoskeleton assessment, past mechanical simulators have not accounted for the effects of soft tissue deformation, leading a mismatch between simulated and human exoskeleton testing outcomes. For example, a knee exoskeleton usually attaches at the thigh and shank. Both limb segments have large volumes of fat and muscle tissue that compress under load. The viscoelastic properties of the soft tissue result in the loss of mechanical energy transfer from the exoskeleton to the user, decreasing the effective assistance transmitted to the joint [17], [18]. A knee simulator that accounts for soft-tissue deformation would provide a more accurate representation of the mechanical energy transfer to the user’s musculoskeletal system.

Few studies have tried to quantify the efficacy of mechanical energy transfer in exoskeletons (i.e., the assistance felt at the joint compared to the device output). Exoskeleton frictional losses and muscle/fat tissue deformation at the human-device interface decrease the assistance to the human joint. Yandell et al. estimated that the interface of an ankle exoskeleton absorbed about 25% of the applied end-effector power and delayed the assistance timing [19]. Cherry et al. found that Bowden cable friction and soft tissue compression together resulted in a ~50% reduction of the expected energy output of their exoskeleton [20]. These studies suggest that soft tissue energy losses can substantially reduce mechanical power assistance compared to the engineering design specifications.

Ballistics gel can simulate the mechanical properties of soft tissue in a human leg simulator for exoskeleton testing. Measurements of elastic modulus, stiffness, and rupture strength suggest that ballistic gel is a good match for human soft tissue. Ballistics gel phantoms have simulated soft tissue in ultrasound training [21], shoulder dislocation training [22], electromyography assessment [23], and lower limb blood flow and pressure [24]. Developing a soft tissue limb simulator for robotic exoskeletons using ballistics gel could provide highly controlled testing dynamics devoid of intersubject variability, and reduce the time and effort in collecting prototype data while reducing the safety concerns of human subject testing.

The purpose of this study is to present a new methodology for quickly testing knee exoskeleton performance and assistance dynamics prior to human subject testing. We achieve this goal by using a novel lower limb simulator consisting of a two-bar linkage while incorporating ballistics gel to simulate soft tissue deformation. We evaluated the lower limb simulator with a passive elastic knee brace to determine how soft tissue deformation contributed to knee exoskeleton assistance losses. We sought to estimate the energy losses from soft-tissue compression during a typical gait cycle by measuring the knee flexion/extension kinetics of the phantom limb with the brace attached to the knee interfacing and bypassing the ballistic gel.

## II. METHODS

### A. Phantom Leg Design

**Fig. 1.**
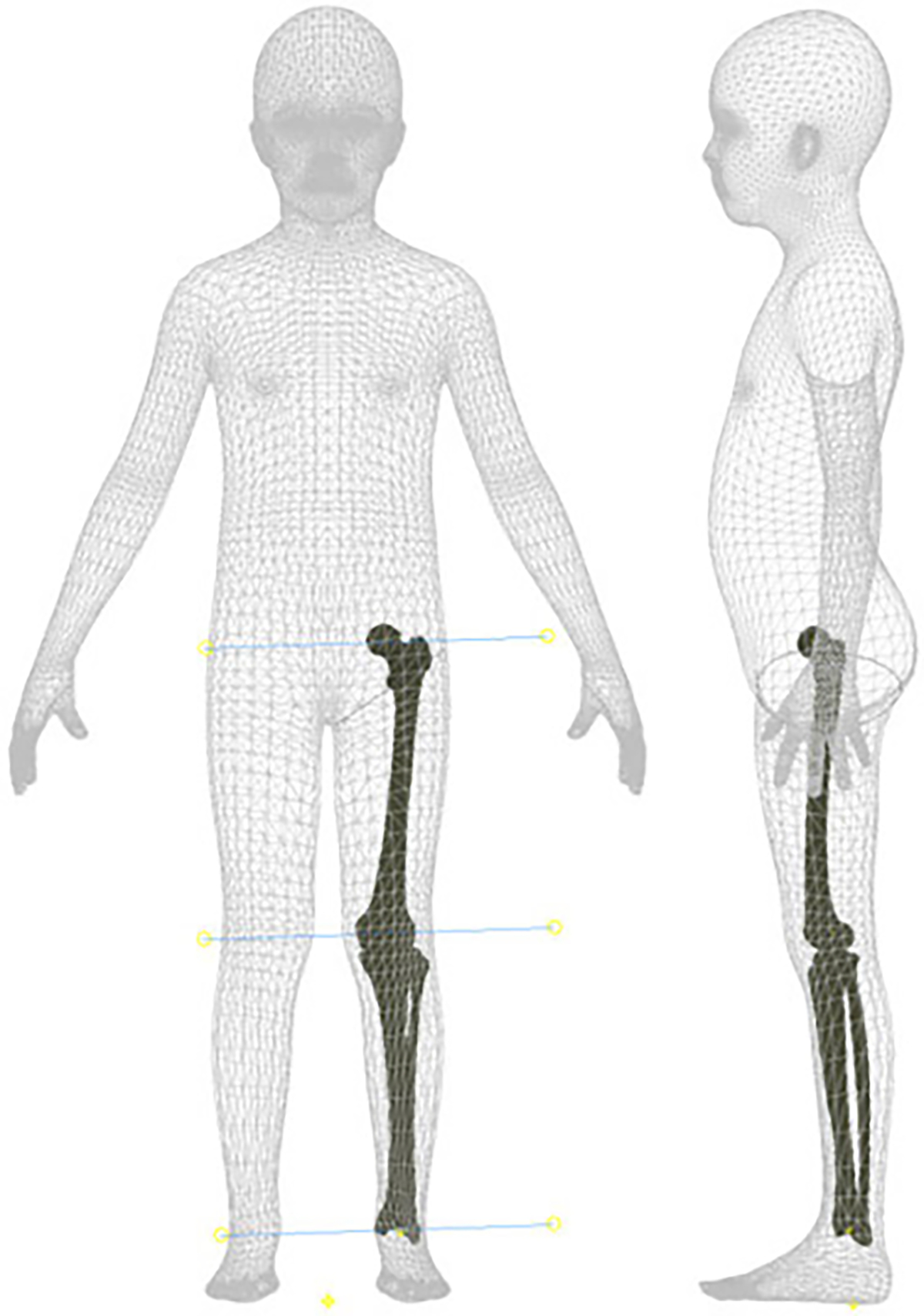
The merged child and bone models used to construct the phantom leg. We used the shown centers of rotation of the ankle, knee, and hip joints to build the mechanical phantom capable of emulating the knee kinematics in the sagittal plane.

We used 3D body and bone models to build a phantom leg the size of a human child’s leg (Fig. 1). The design goal was a child sized phantom because future work in our laboratory will use the phantom limb to examine the efficacy of a child-size lower limb exoskeleton for walking. We downloaded a standing child model avatar from the online BioHuman repository (male, 1.31m height, $17\;kg/m^{2}$ body mass index, 0.53m sitting height to stature ratio) [25]. Using Autodesk Inventor 2021, we isolated the left leg and split it into thigh and shank segments. Similarly, 3D meshes of the femur, tibia, and fibula were downloaded from The Living Human Digital Library [26]. Using Horn’s method [27], [28], we optimized the bone scaling, rotation, and translation to fit the body landmarks of the child model.

We further modified the leg-bone model before assembling the final design. The flexion-extension axis of the knee was approximated with the cylinder axis, the line connecting the best-fitted spheres of the lateral and medial femoral condyles, as described previously by Yin et al. [29]. Using the same axis of rotation for the ankle and hip joints allowed us to move the leg in the sagittal plane. The bones were 3D-printed with Onyx filament and reinforced with continuous carbon fiber using a Markforged X7 3D Printer (Markforged Inc., Watertown, MA, USA). We also 3D-printed molds of the thigh and shank to surround the bones with 20% ballistic gelatin (ballistic powder weight per water volume). Ball bearings at each joint allowed us to assemble and rotate the hip, knee, and ankle joints of the leg with minimal friction.

### B. Control and Instrumentation

We designed the phantom leg to simulate most sagittal human knee trajectories. Fixing the hip to an overhead support and moving the ankle vertically with a Notus hexapod (Symetrie, Nîmes, France) allowed us to move the leg through passive coupling of the joints.

**Fig. 2.**
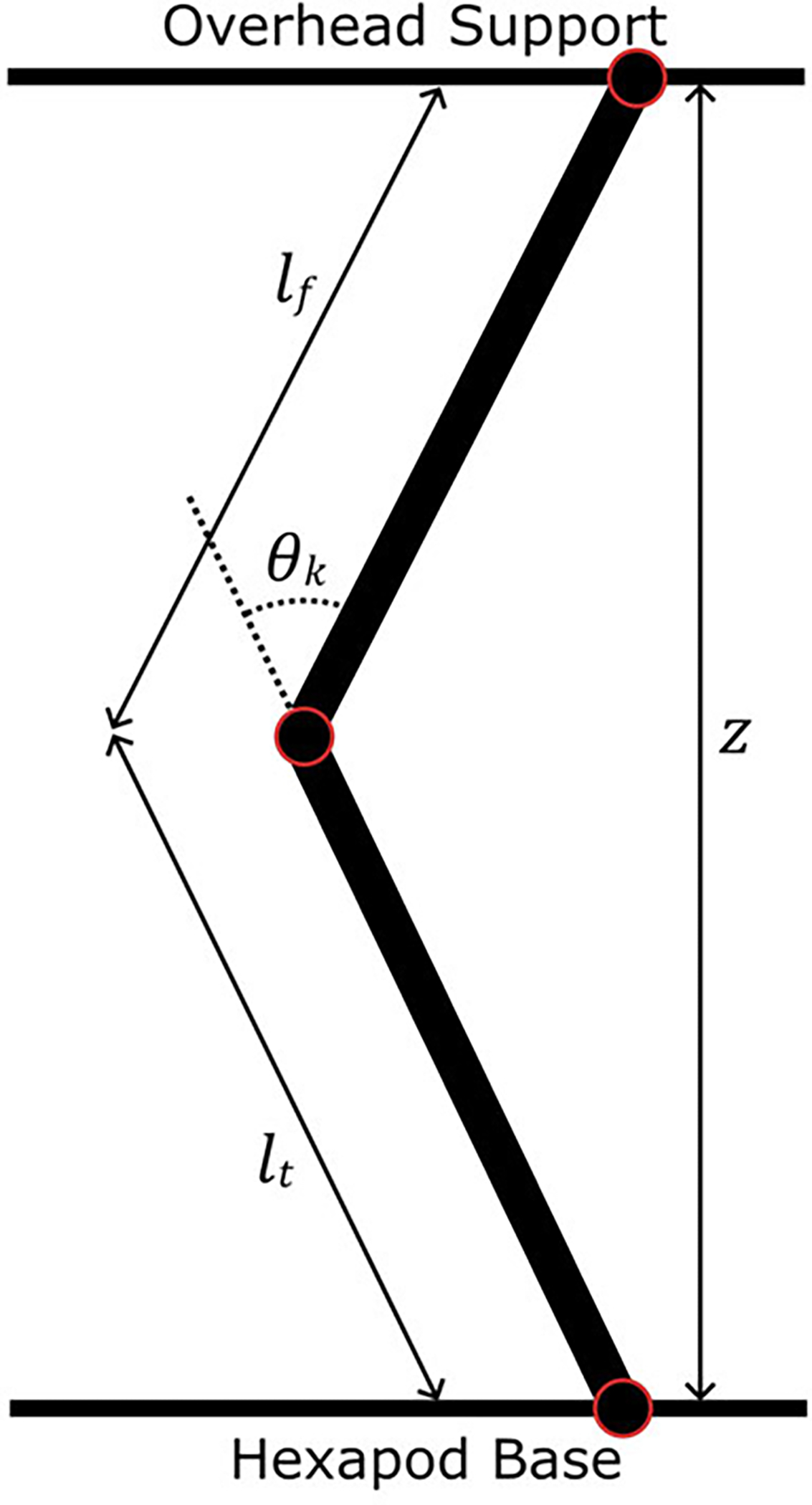
The phantom limb modeled as a two-bar linkage with bar lengths $l_{f}$ and $l_{t}$. The hexapod base moved vertically to change the vertical height z and achieve a desired knee angle $\theta_{k}$.

A custom MATLAB program computed the height of the hexapod base relative to the hip to reach the desired knee angle (Fig. 2). Placing the hip joint directly on top of the ankle, we calculated the vertical distance z from the hexapod base to the overhead support, given the known variables femur length $l_{f}$, tibia/fibula length $l_{t}$, and knee angle $\theta_{k}$ (1). As a result, rising and lowering the hexapod base flexed and extended the knee joint in the sagittal plane correspondingly. The phantom could flex the knee to 81°, at which point the ballistic gel of the thigh and shank would collide. A locking mechanism stopped the knee from extending lower than 2° to prevent the mechanical singularity where the hip, knee, and ankle axes are colinear.


(1) $$z^{2}=l_{f}^{2}+l_{t}^{2}-2l_{f}l_{t}\;cos\left(\pi -\theta_{k}\right)$$

We instrumented the phantom leg to provide force and motion readings for inverse dynamics calculations. Three REB7 compression/tension load cells (Loadstar Sensors, Fremont, CA, USA) placed under the ankle measured the vertical reaction forces at 1000 Hz. Four OptiTrack cameras recorded the positions of markers attached to the medial and lateral sides of the hip, knee, and ankle joints at 100 Hz.

### C. Inverse Dynamics Model

**Fig. 3.**
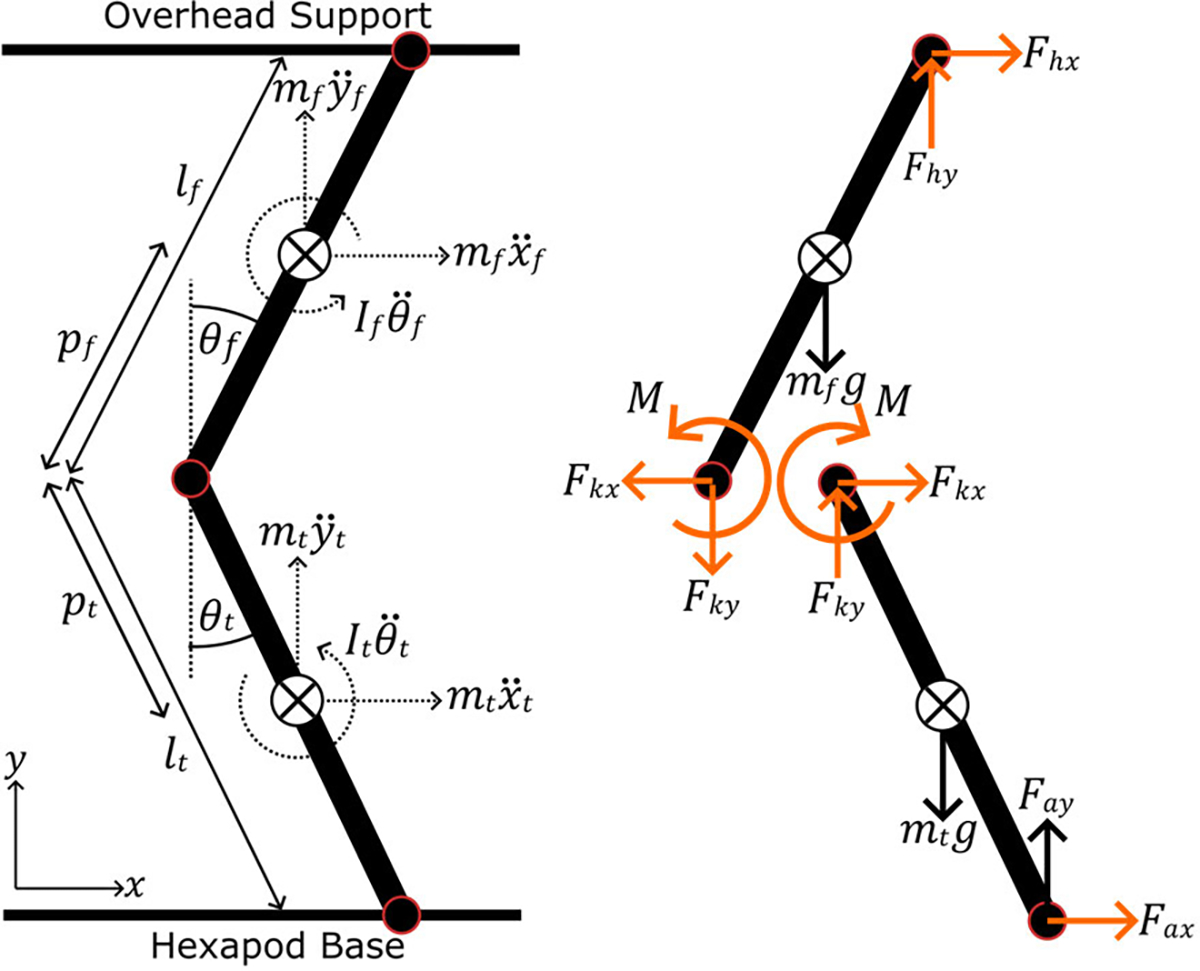
The inertial and internal forces and moments of the phantom limb. The inverse dynamics model estimates the moment at the knee and other unknown variables (highlighted orange) from known quantities linkage weight, inertia, length, linear/angular accelerations, and load cell forces.

We used a custom inverse dynamics model to estimate the moment provided to the knee by any knee exoskeleton. The model approximates the leg as a two-bar linkage moving only in the sagittal plane. Fig. 3 shows the inertial and internal forces of the femur and tibia/fibula linkages. Assuming negligible joint friction, we built a dynamics system containing six unknown internal forces, including the knee moment. Applying Newton’s second law yields three equations of motion per linkage for a total of six independent equations to solve. Equation (2) shows the matrix form of the equations that we solved simultaneously in MATLAB to estimate the knee moment of the phantom at every experimental time point. Table I presents the model variables in more detail. We estimated the body segment parameters (center of mass position and moment of inertia) of both linkages from the Autodesk Inventor assembly and experimentally-measured building material densities.


(2) $$\begin{array}{c} \left[\begin{array}{cccccc} 0 & 0 & 1 & 0 & 0 & 0\\ 1 & 1 & 0 & 0 & 0 & 0\\ l_{t}\;cos\;\left(\theta_{t}\right) & 0 & 0 & 0 & 0 & -1\\ 0 & 0 & -1 & 0 & 1 & 0\\ 0 & -1 & 0 & 1 & 0 & 0\\ 0 & 0 & 0 & -l_{f}\;cos\left(\theta_{f}\right) & l_{f}\;sin\left(\theta_{f}\right) & 1 \end{array}\right]\left[\begin{array}{c} F_{ax}\\ F_{kx}\\ F_{ky}\\ F_{hx}\\ F_{hy}\\ M \end{array}\right]\\ =\left[\begin{array}{c} -F_{ay}+gm_{t}+m_{t}{\ddot{y}}_{t}\\ m_{t}{\ddot{x}}_{t}\\ gm_{t}{\;p}_{t}\;sin\left(\theta_{t}\right)-F_{ay}l_{t\;}sin\left(\theta_{t}\right)+l_{t}{\ddot{\theta }}_{t}\\ gm_{f}+m_{f}\ddot{y_{f}}\\ m_{f}\ddot{x_{f}}\\ gm_{f}{\;p}_{f}\;sin\left(\theta_{f}\right)-I_{f}\ddot{\theta_{f}} \end{array}\right] \end{array}$$

### D. Phantom Loading

We programmed the hexapod to flex and extend the knee of the phantom following sample gait kinematics of children 7–12 years old walking at 1 m/s [30]. Equation (1) estimated the hexapod height relative to the hip joint to reach the desired knee angles. The sample data was initially normalized to gait cycle percentage but was interpolated to obtain a 1 s stride time sampled at 100 Hz. We exported the resulting hexapod trajectory to the SYM_Motion software (Symetrie, Nîmes, France) to execute the respective hexapod motions and move the knee through multiple continuous gait cycle trajectories. We recorded marker and load cell data for each loading condition trial.

To assess the effects of soft tissue deformation on knee assistance throughout the gait cycle, we loaded the joint with torsional springs under two conditions: interfacing (brace-loading) and bypassing (braceless-loading) the ballistic gel. We chose to load the knee with torsional springs because a healthy human knee has a linear moment-angle relationship in the stance phase and an exoskeleton assisting the knee in stance would provide a linear moment with respect to flexion angle [31]. Although the swing phase has a non-linear moment, we decided to keep the springs engaged throughout the entire gait cycle for device simplicity.

**Fig. 4.**
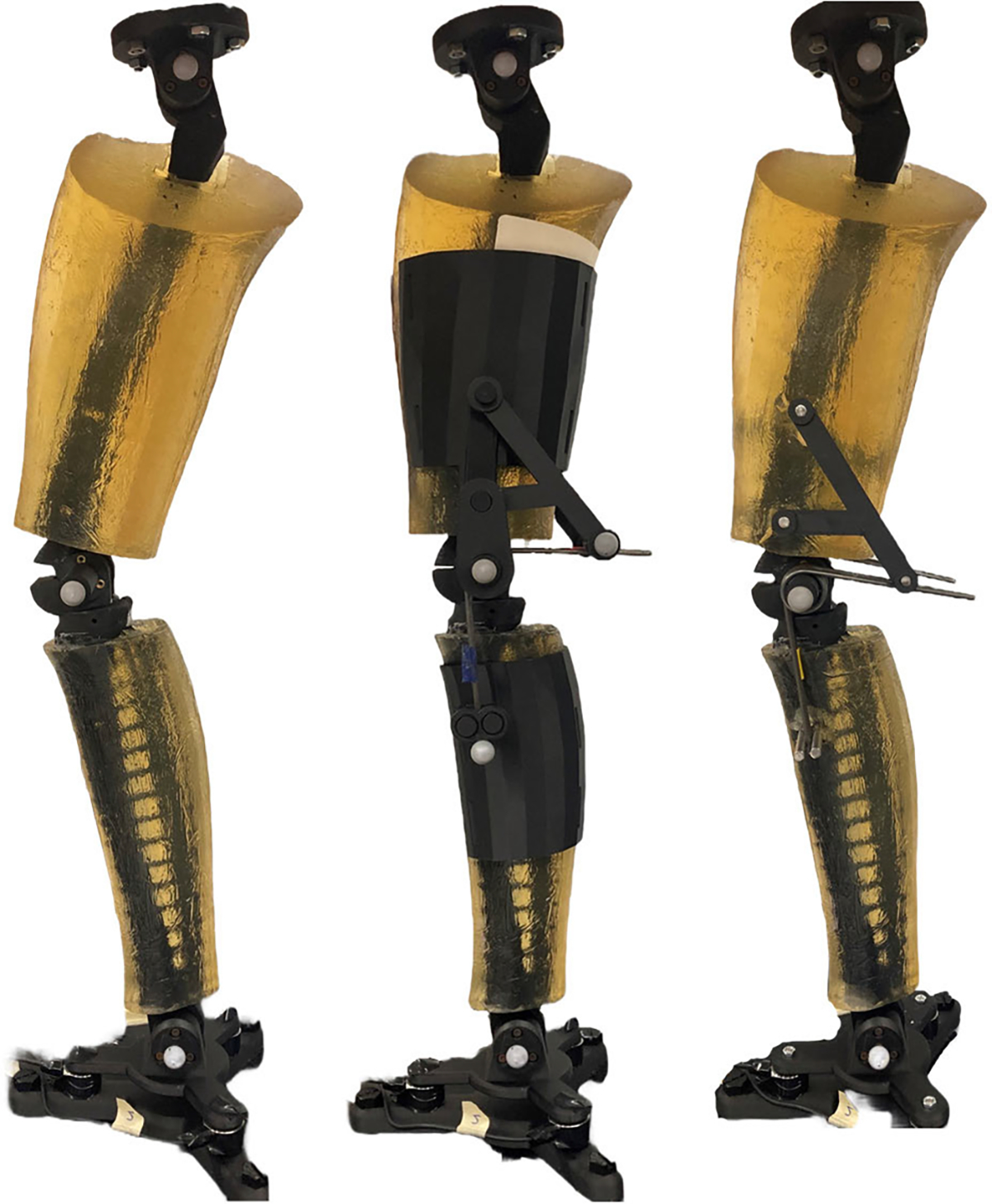
The phantom lower limb mounted on the hexapod under various loading conditions. The left image presents the phantom without any springs or braces. Middle image shows the phantom with torsional springs attached to braces interfacing the ballistic gel of the thigh and shank. The right image shows the phantom with the same springs bypassing the ballistic gel and attaching directly to the femur and tibia. Load cells attached under the ankle and marker positions at the joints are used to estimate the knee moment.

The brace-loading condition had the torsional springs connected to 3D-printed braces interfacing the ballistic gel of the thigh and shank (Fig. 4). We designed and placed the braces to load the springs throughout the entire gait cycle with a slight spring pre-tensioning at full knee extension. Two torsional springs centered at the knee were placed at the medial and lateral sides of the leg. We attached the two ends of the springs to the thigh and shank braces and the spring coil around the dowel pin of the knee. The resulting assembly flexed the torsional springs when the hexapod raised the ankle, creating an assistive moment estimated by the inverse dynamics model. Preliminary loading tests resulted in gel tearing near the edges of the thigh brace and led us to cover the thigh with artificial silicone skin for increased tear resistance. Additionally, a linkage connecting the thigh brace to the knee dowel pin prevented upwards slipping of the brace from the torsional spring forces. In addition to the joint markers, reflective markers were attached to the spring ends to estimate and compare the spring deflection angle to the knee angle.

**Fig. 5.**
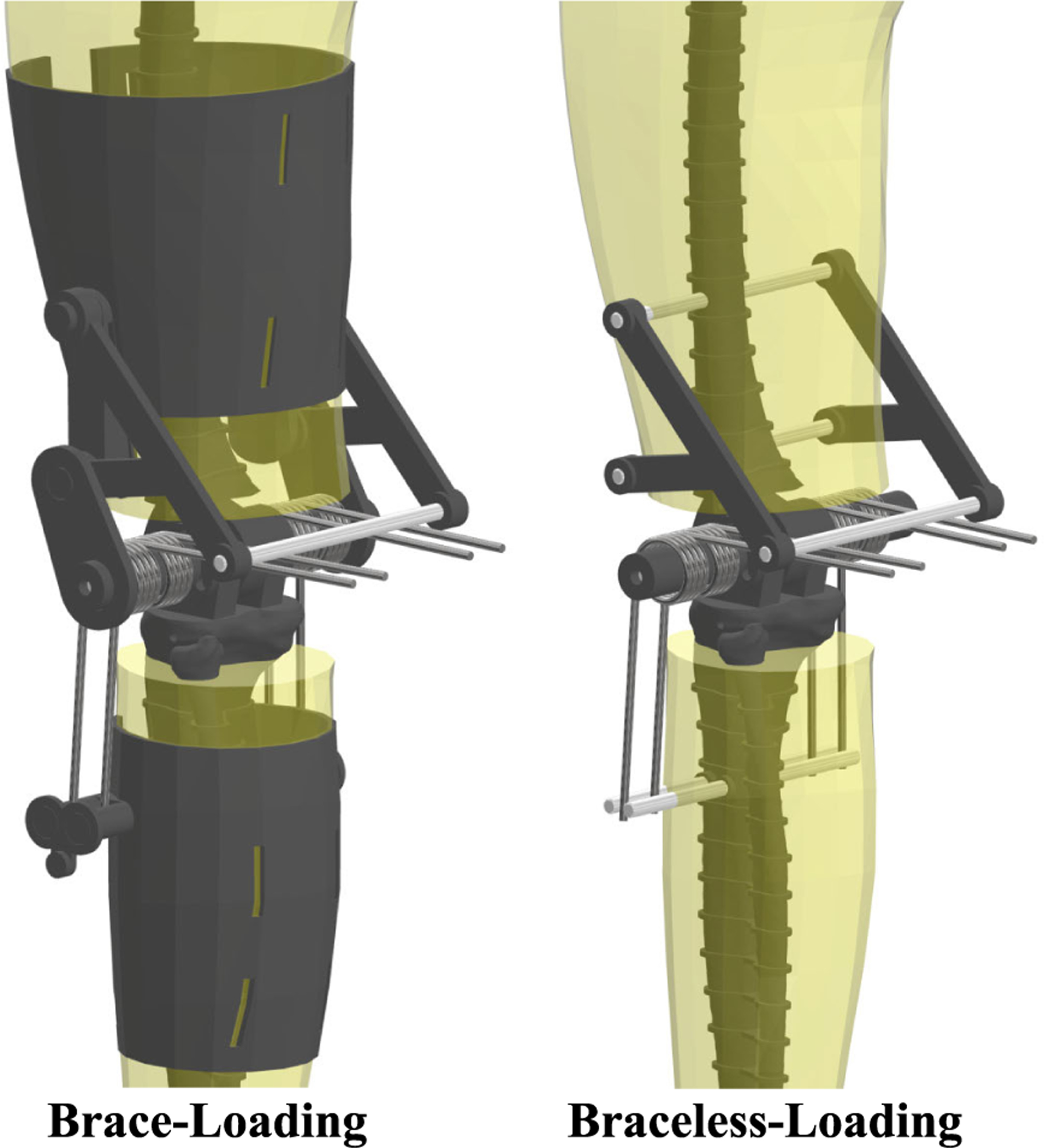
Detailed view of the of the phantom lower limb for both loading conditions. Four torsional springs centered about the knee joint deflect with knee flexion to create an extensor joint moment. In brace-loading, the ends of the torsional springs connect to thigh and shank braces interfacing the ballistic gel of the phantom. In braceless-loading, the spring ends connect directly to the bones, bypassing the ballistic gel.

The braceless-loading condition had the same torsional springs bypassing the ballistic gel and attaching directly to the femur and tibia bones. We attached the ends of the springs to pins going through the femur and tibia (Fig. 5) so that full knee extension corresponded to zero spring angular deflection.

### E. Spring Characterization

We characterized the mechanical properties of the springs to compare with our inverse dynamics model estimates of spring stiffness. Each spring was individually loaded under torsion on an 858 Mini Bionix II (MTS Inc., Eden Praire, MN, USA) over a triangle wave of 45° amplitude and 1 Hz frequency. The spring moment and angle were recorded at 99.9 Hz and exported to MATLAB for further processing.

### F. Data Processing

We used MATLAB to process the raw data and estimate the knee moment provided to the joint by the torsional springs. The marker and load cell data were filtered using a fourth-order zero-phase Butterworth filter with a 6 Hz cut-off frequency. The marker data was interpolated to 1000 Hz using a spline function to match the load cell data frequency. After projecting to the lower limb sagittal plane, the midpoint between each joint’s medial and lateral markers provided the joint centers of rotation, knee angle, and linear/angular accelerations of both phantom linkages. The root-mean-square error (RMSE) was estimated among both phantom loading conditions and the input knee kinematics to quantify tracking performance. We converted the voltage readings of the three load cells to force and added them to estimate the vertical reaction force at the ankle. Using Autodesk Inventor, we estimated the inertia and center of mass locations for each phantom condition (Table II). Equation (2) calculated the knee moment at every time point of both phantom loading conditions from the estimated body segment parameters, inertial forces, and load cell forces.

We used angle-moment profiles to provide more insight into the exoskeleton assistance. We plotted the knee moment estimated from inverse dynamics relative to the knee angle for both brace and braceless loading conditions. Additionally, we plotted the same knee moment with respect to the spring deflection angle to assess the effects of soft tissue deformation on joint assistance. The spring deflection angle was estimated from the knee and spring-end markers for the brace condition, and we assumed that the spring angle was equal to the knee angle in the braceless condition. A linear regression of the work loops of each phantom condition calculated the angular stiffness provided to the joint. We calculated the work hysteresis as the area under the curve of the work loop plot relative to the total positive work to estimate the energy lost during the knee loading and unloading phases.

We processed data from the spring torsion tests to estimate the cumulative stiffness of the four torsional springs. We filtered the recorded moment and angle data using a fourth-order zero-phase low-pass Butterworth filter with a 10 Hz cut-off frequency. A linear regression estimated the stiffness of each spring, which we use to calculate the cumulative spring stiffness.

### G. Gel Stiffness Assessment

We used a MyotonPro (MyotonAS, Tallinn, Estonia) to assess the mechanical properties of different ballistic gel densities at different temperatures. The viscoelastic properties of ballistic gel are highly dependent on its temperature [32]. The MyotonPro is a reliable device capable of quantifying the viscoelastic properties of superficial muscles, tendons, skin, and subcutaneous fat [33]. The device applied a pre-pressure force (0.18 N) through an indentation probe (∅3mm) to the ballistic gel before pushing the probe with a mechanical impulse (0.4 N, 15 ms). A built-in accelerometer recorded the indentation deflection over time to calculate the dynamic stiffness of the gel. We prepared cylindrical ballistic gel samples (∅100mm×50mm) of different densities (10%, 12.5%, 15%, 17.5%, and 20%) and measured their dynamic stiffness in roughly 30 min intervals after they were taken out of the fridge until reaching at least 20 °C. We took five consecutive MyotonPro measurements to estimate the dynamic stiffness of the gels at each time point.

## III. RESULTS

### A. Tracking

**Fig. 6.**
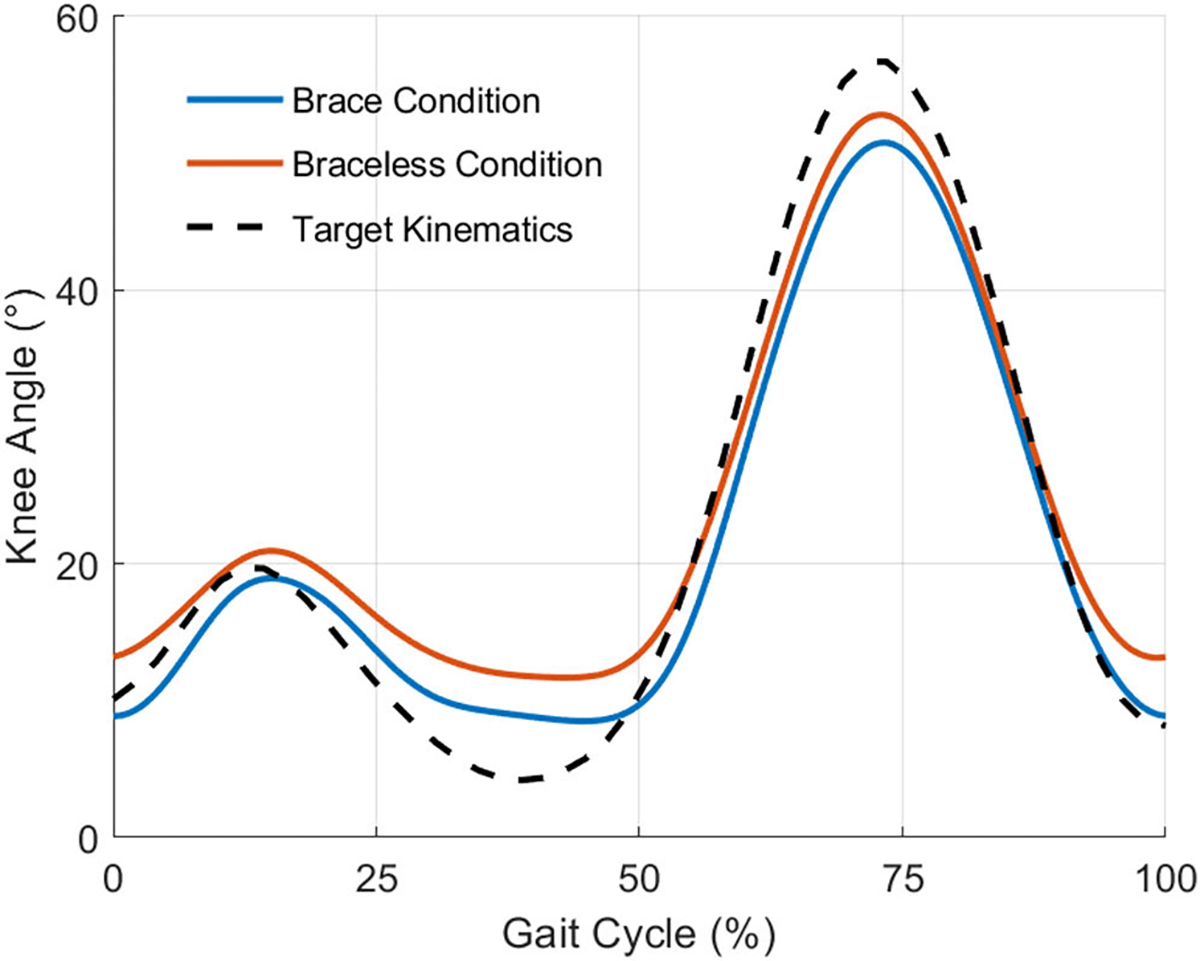
The mean knee angle of 15 gait cycles for both phantom loading conditions and the target knee kinematics. Tracking errors were present compared to the input kinematics and were the largest at maximum knee flexion and extension.

We plotted the gait cycle kinematics and calculated root-mean-square error (RMSE) between both phantom loading conditions and the target kinematics. Although small, tracking errors were present in both phantom trajectories compared to the target knee kinematics (Fig. 6). Across the entire gait cycle, the RMSE of the brace and braceless conditions with respect to the target were 3.6° and 4.1° respectively (Table III). From Fig. 6, tracking error was biggest at the instances of maximum knee flexion and extension.

### B. Knee Moment Profile

**Fig. 7.**
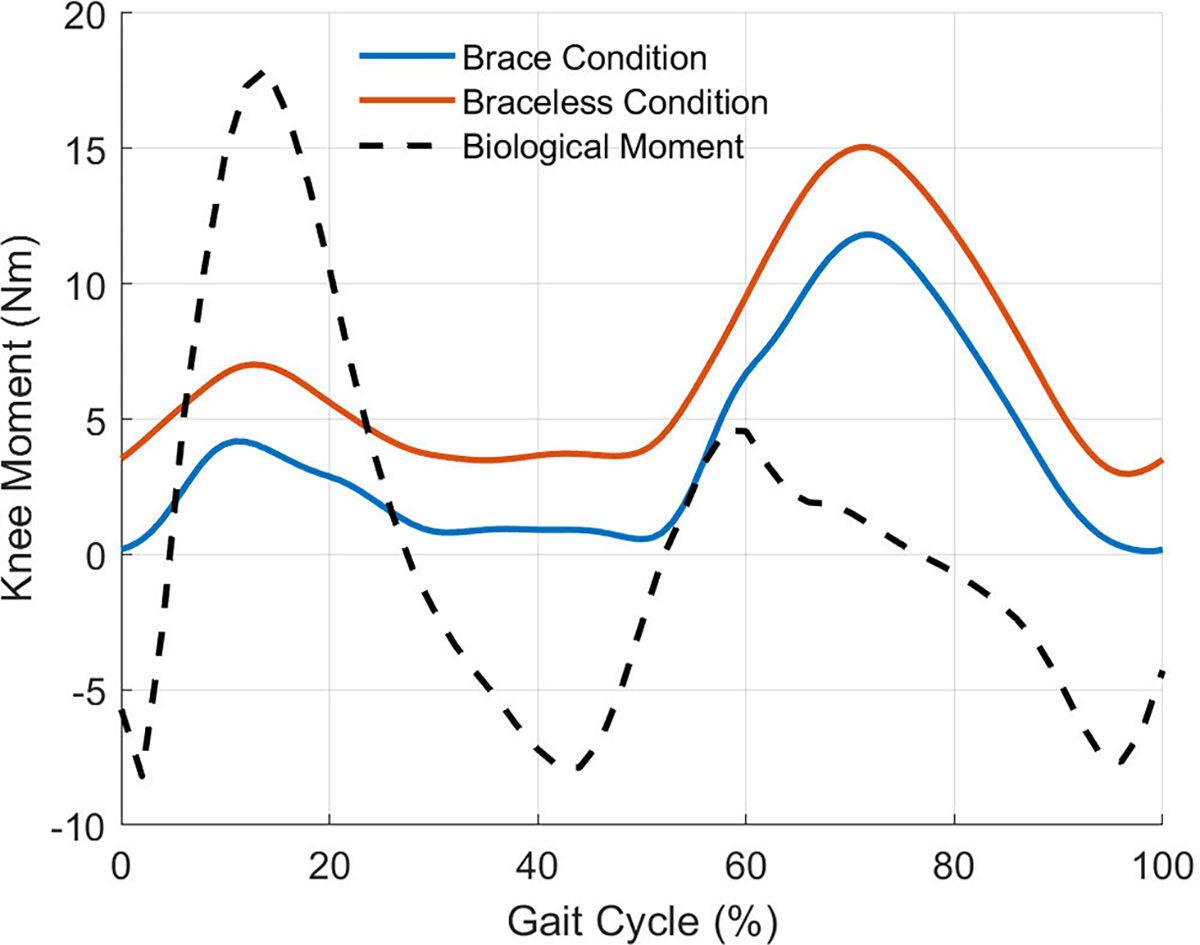
The mean knee moments provided by the passive elastic exoskeleton throughout 15 gait cycles in comparison to the expected biological moment of a real human knee. Braceless-loading provided greater knee moments at every gait instance compared to brace-loading.

**Fig. 8.**
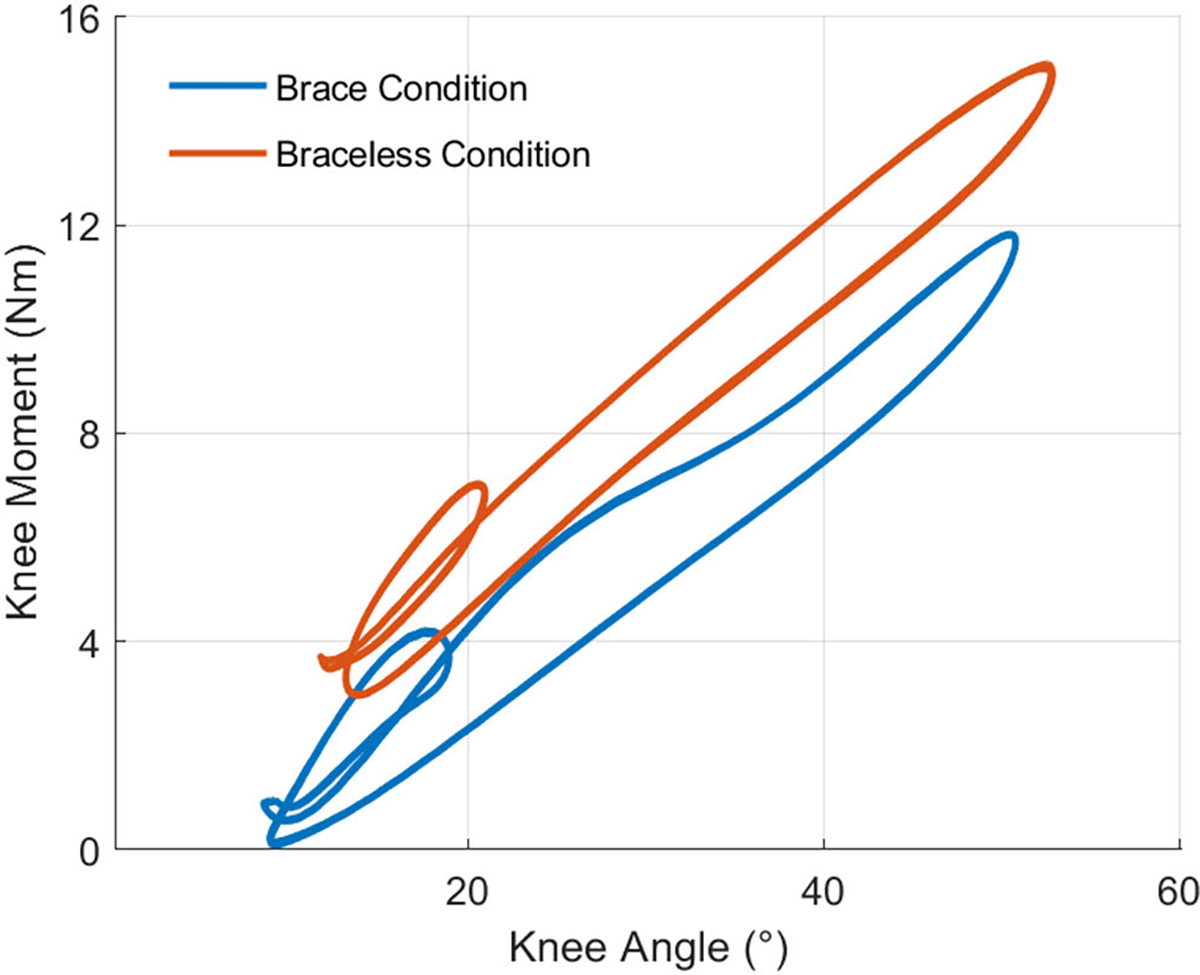
Work loops of 15 gait cycles showing the relation between knee moment and knee angle for both phantom loading conditions. Each work loop contained two inner loops corresponding to the stance and swing phases of gait. The assistance profile of brace-loading, interfacing the ballistic gel, had an offset and lower angular stiffness relative to braceless-loading, bypassing the gel.

For both phantom loading conditions, we plotted and analyzed the work loops obtained from the phantom knee moment (Fig. 7) and angle trajectories (Fig. 6) over 15 gait cycles. Fig. 7 shows the moments generated by the passive elastic exoskeleton in comparison to the expected biological knee moment of a child the same size as the phantom model walking with a natural cadence [34]. The work trajectories over one gait cycle contained two inner loops corresponding to the loading and unloading phases of stance and swing (Fig. 8). From linear regression models (Table IV), the torsional springs provided a higher angular stiffness when bypassing the ballistic gel in braceless-loading (15.9± 0.01 Nm/rad) than when interfacing it in brace-loading (14.6 ± 0.02 Nm/rad). Besides a lower stiffness, loading through the braces also resulted in a ~6.77° x-Intercept offset, further contributing to a lower assistance moment at any given knee angle. The hysteresis, calculated as the area under the curve (AUC) normalized to the total positive work, estimated the energy lost during the loading and unloading phases. As expected, the AUC was higher when loading through the ballistic gel (0.24) than loading directly to the bone (0.15).

### C. Adjusted Knee Moment Profile

**Fig. 9.**
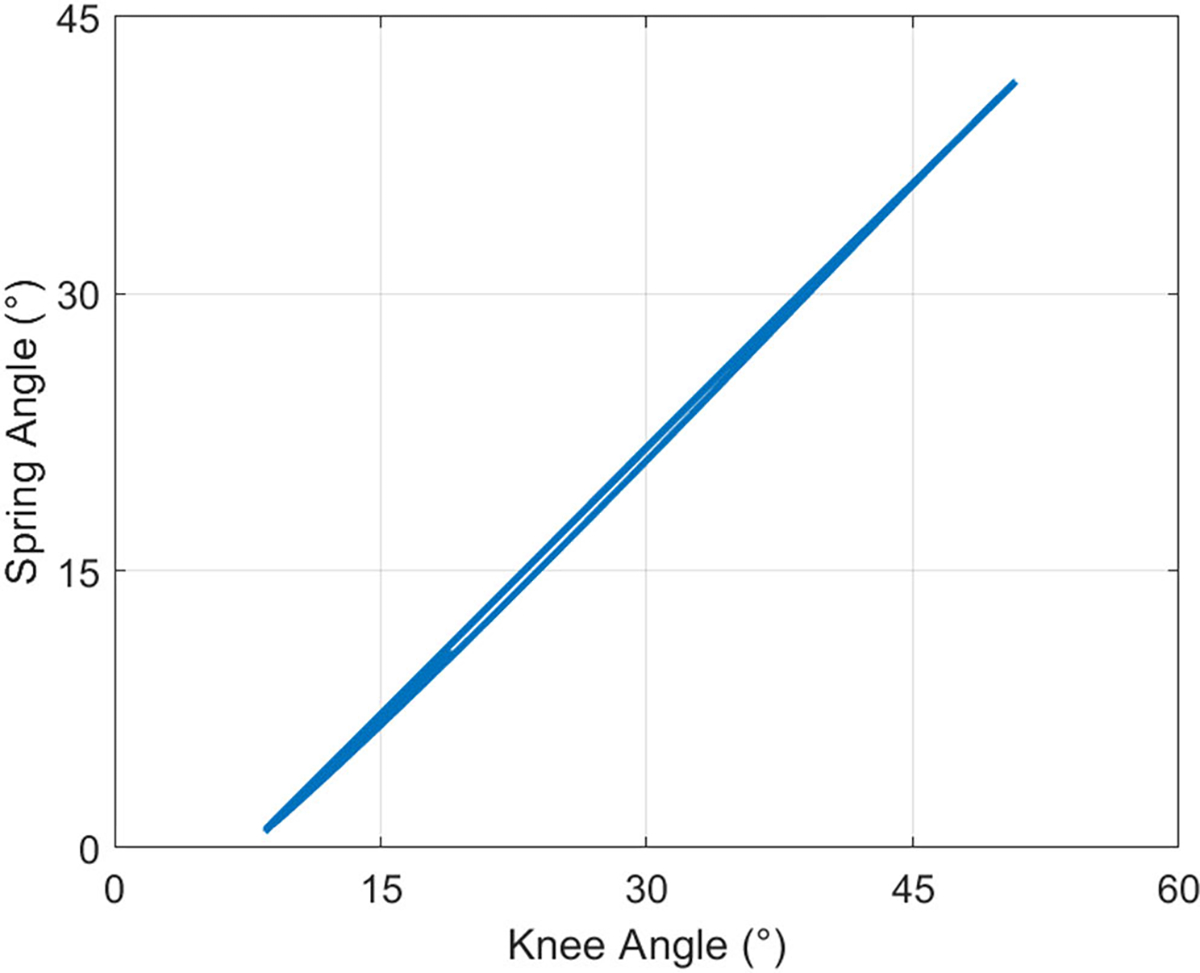
Spring angle as a function of knee angle in the brace-loading condition for 15 gait cycles. The spring angle was lower than the knee angle for all time points, contributing a lower assistance knee moment.

We used spring markers to assess the effects of soft-tissue deformation on the assistance profile. In brace-loading, plotting the spring angle $\theta_{s}$ as a function of knee angle $\theta_{k}$ revealed a strong linear relationship between the variables (Fig. 9). The resulting linear regression $\left(\theta_{s}=0.96\theta_{k}-7.4^{\circ }\right)$ showed that the springs had a 7.4° offset at full knee extension and increased with respect to the knee angle by a factor of 0.96.

**Fig. 10.**
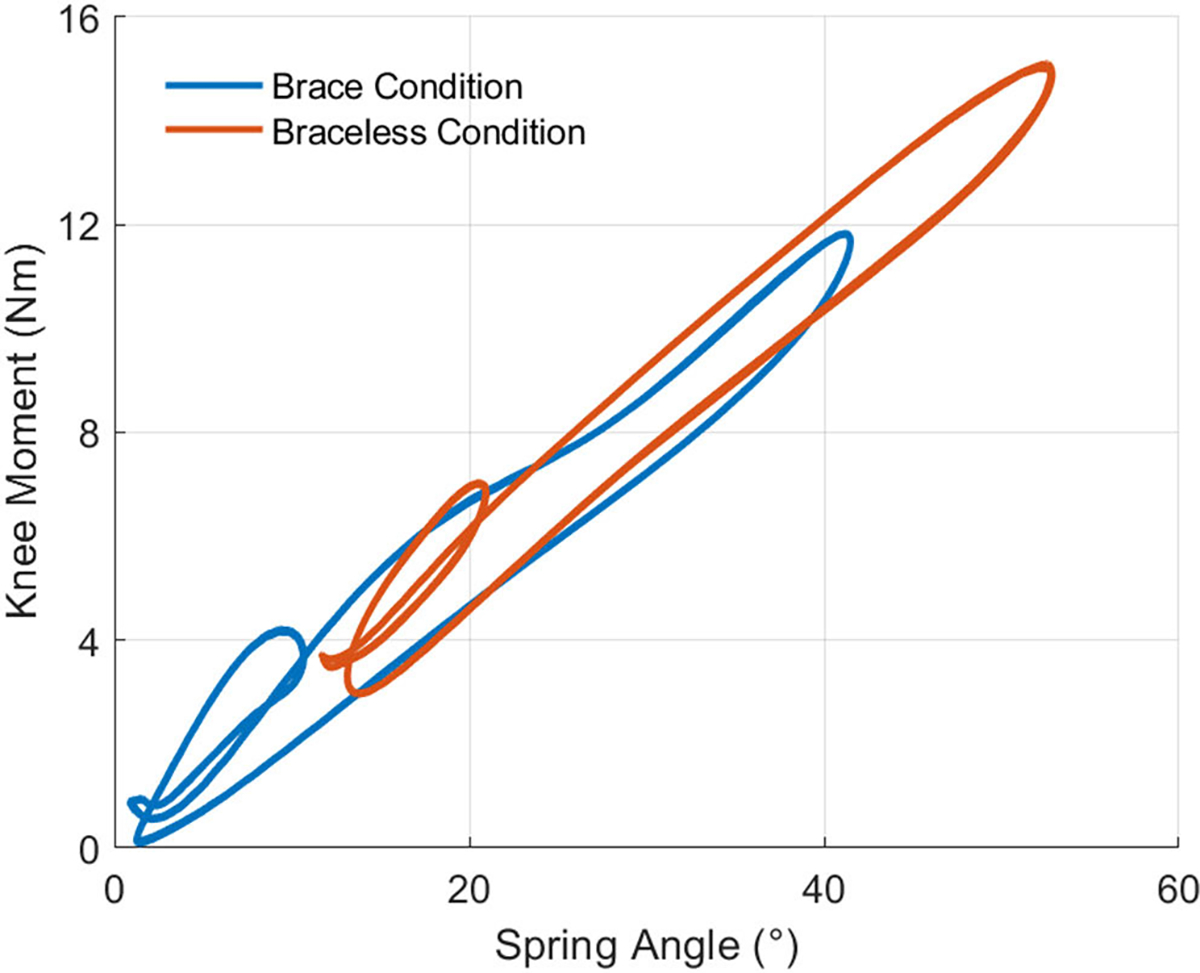
Phantom assistance profiles adjusted for the spring angle for 15 gait cycles. The linear regression values of stiffness and offset of the brace-loading condition were closer to those of braceless-loading. The AUC hysteresis was mostly unaffected by the spring angle adjustment.

We plotted the knee moment as a function of the spring angle for the same 15 gait cycles to assess whether previous assistance profile differences could be attributed to differences in the spring angle. As described previously, we assumed that the spring angle was equal to the knee angle in braceless-loading bypassing the ballistic gel. Fig. 10 shows that the brace-loading assistance profile followed the braceless-loading condition more closely. The assistance slope of the brace loading condition was larger (15.2 ± 0.01 Nm/rad) than previously estimated when corrected with the spring angle but still slightly lower than the braceless-loading stiffness (15.9 ± 0.02 Nm/rad) (Table V). The AUC hysteresis of the brace condition only decreased by 0.01 when accounting for the spring deflection. Lastly, the offset of both linear regression models corrected for spring deflection was reduced to ~0.884°.

### D. Spring Characterization

**Fig. 11.**
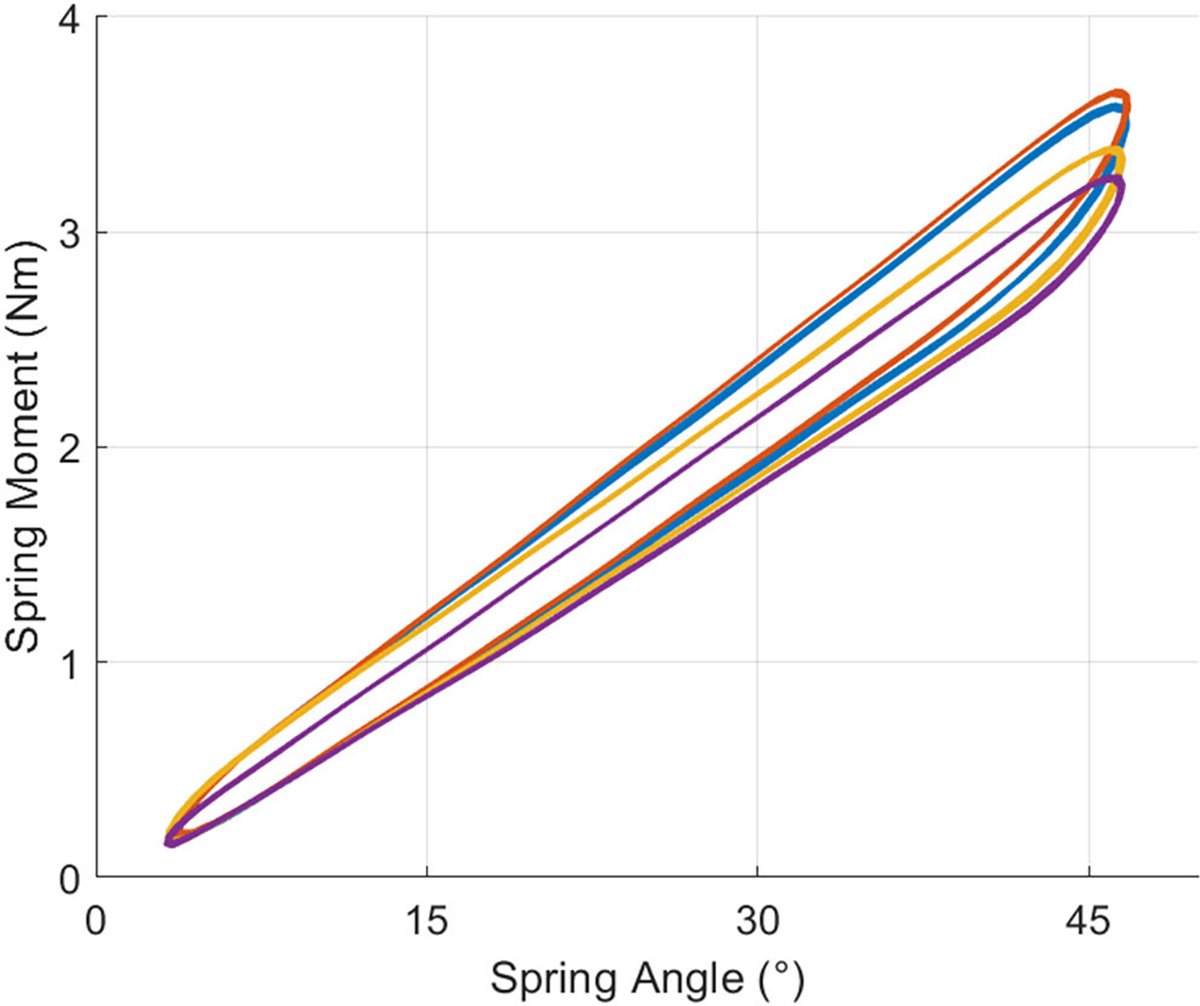
Torsional test results of each of the four springs used to load the phantom knee. All spring moments showed a linear relationship with the spring angle. The cumulative spring stiffness estimated from braceless-loading inverse dynamics was within ~4.2% of the cumulative torsional spring stiffness derived from the torsional tests.

We estimated the cumulative spring stiffness derived from torsional tests to assess the accuracy of our inverse dynamics model. Fig. 11 shows the moments and angles of 14 cycles of torsional spring loading and unloading for each of the four springs attached to the phantom knee. A linear regression model estimated the cumulative spring stiffness to be 16.6 ± 0.01 Nm/rad. The inverse dynamics model of braceless-loading bypassing the ballistic gel calculated a cumulative spring stiffness (15.9 ± 0.02 Nm/rad) ~4.2% lower than that estimated from mechanical torsion tests.

### E. Gel Stiffness Assessment

**Fig. 12.**
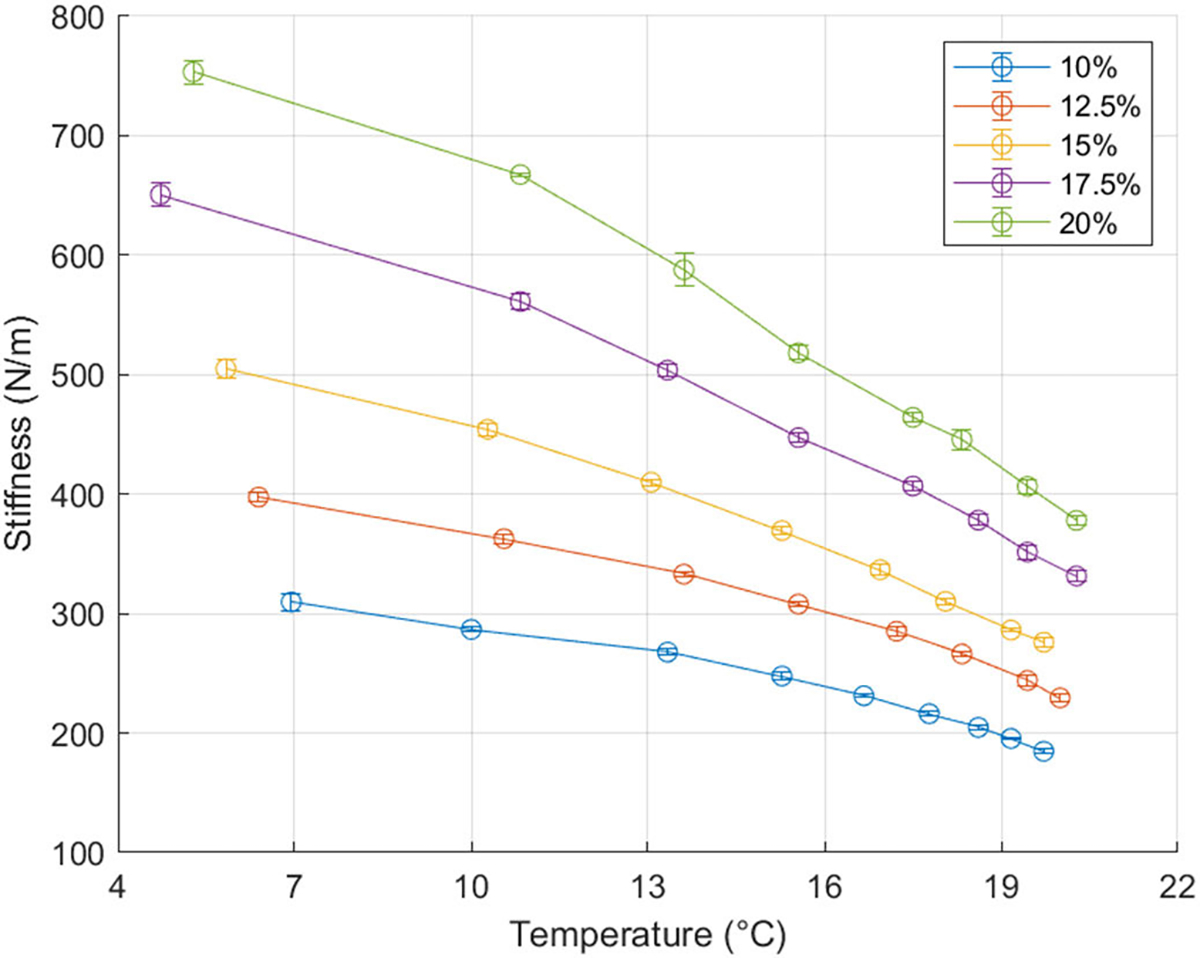
Ballistic gel dynamic stiffness over a range of gel densities and temperature. Gel stiffness increased with increasing ballistic gel density (ballistic powder weight per water volume) and decreased with increasing temperature. The error bars represent the standard deviation of five MyotonPro dynamic stiffness measurements on a single sample.

We estimated the dynamic stiffness of the ballistic gel over a range of gel densities and temperatures. Fig. 12 shows the dynamic stiffness of the gels taken at roughly 30 min intervals after being taken out of the fridge. As expected, stiffness increased with ballistic gel density at any given temperature. Similarly, stiffness decreased with increasing temperature for all ballistic gel samples.

## IV. DISCUSSION

We have introduced a new methodology for quickly testing and validating the performance of knee exoskeletons prior to human subject testing. Our lower-limb mechanical phantom can simulate knee kinematics while accounting for the soft-tissue deformation at the exoskeleton interface. Our experimental results suggest that soft-tissue deformation and energy dissipation at the exoskeleton-phantom interface lead to a lower assistance moment transmitted to the joint. The novel phantom limb framework will be useful for future exoskeleton development and prototyping.

Tracking performance of the mechanical phantom was good but not perfect due to the open loop control. The tracking error was most noticeable in instances of maximum knee flexion and extension. Our phantom had a maximum RMSE of 4.1° relative to its target kinematics. However, it is not uncommon to have an intersubject mean knee angle standard deviation of 5.3° between individuals walking at the same cadence [34]. A future iteration of the phantom may implement a closed-loop control that tracks the output kinematics to reduce errors in joint kinematics. Concerning the intersubject variability, our initial efforts of phantom motion are not unreasonable. Given the linear nature and stiffness of the springs used in our testing, we do not believe the kinematic variability to have altered the main conclusions from our testing.

To assess the effects of soft-tissue deformation on exoskeleton assistance, we loaded the knee with torsional springs under two conditions, through a brace interfacing the ballistic gel (brace-loading) and directly to the bone (braceless-loading). Using inverse dynamics, we found that brace-loading resulted in a significantly lower knee assistance stiffness of 14.5 ± 0.01 Nm/rad compared to the braceless-loading condition of 15.9 ± 0.02 Nm/rad. Accounting for soft-tissue deflection, brace-loading provided a 15.2 ± 0.01 Nm/rad to the joint, still lower than braceless-loading. We found that ~50% of the difference in knee stiffness across loading conditions could be attributed to gel deflection. Like adipose and muscle tissue, ballistic gelatin has viscoelastic properties [32] that likely contributed to energy dissipation and a lower assistance moment to the joint. Besides the gel, the 3D-printed braces could have also deformed and contributed to energy dissipation of the system. However, we suspect their effect to be negligible relative to the gel as they were reinforced with carbon fiber. Besides assistance stiffness, the gel deformation led to a lower spring deflection angle relative to the knee. Our linear regression model $\left(\theta_{s}=0.96\theta_{k}-7.4^{\circ }\right)$ indicated that at full knee extension, ballistic gel compression led the braces to hyperextend by 7.4° relative to the knee angle. Subsequent flexion of the phantom knee showed that the brace angle rate of change was smaller than the knee angle rate of change by a factor of 0.96. We suspect that the initial brace hyperextension was caused by spring loading as we pre-tensioned the springs at full knee extension to ensure engagement of the springs throughout the gait cycle. The rate of change differences can be attributed to the elastic properties of the gel, which compressed under loads and resulted in an increasing difference between brace and knee angle. Although small, these results are dependent on spring stiffness and we suspect that a higher spring stiffness will lead to greater deviations of the brace angle relative to the knee angle. Nevertheless, our results are comparable to previous literature values as Cherry et al. found that a 55° knee angle corresponded to a 47.5° elastic orthosis angle, a 7.5° difference resulting from soft-tissue deformation [36]. We calculated the hysteresis as the area under the curve of the knee moment vs. angle work loops to quantify the energy dissipated between loading and unloading phases of the device. Braceless-loading bypassing the ballistic gel resulted in a 15% hysteresis, likely caused by the inherent hysteresis of the springs, load cells, and 3D-printed bones. Adding the brace-gel interface increased the hysteresis to 24%, which was barely reduced to 23% by accounting for brace deflection. These results suggest that the viscoelastic properties of ballistic gel led to energy absorption and dissipation in the loading and unloading phases. Again, these results will vary for different exoskeleton interface designs.

The custom inverse dynamics model performed well, given our assumptions and approximations. We expected the cumulative spring stiffness, estimated from spring torsion tests, to equal the braceless-loading assistance stiffness at the knee, estimated from inverse dynamics. However, the latter (16.6 ± 0.01 Nm/rad) was higher than the former (15.9 ± 0.02 Nm/rad) by 4.2%. Inverse dynamics estimates are prone to errors from body segment parameter estimates, joint center inaccuracies, skin movement artifacts, sensor hysteresis, and noise [37]. Our phantom has markers attached directly to the bone and is thus not affected by skin movement artifacts. However, the vertical acceleration of the load cells (not experienced by conventional force plates) could have affected sensor readings. Other sources of error include out-of-plane forces, ankle and hip frictional moments not captured by the model, and off-axis load cell loading. Nevertheless, our custom model error falls below the 6% minimum uncertainty of conventional lower-limb human inverse dynamics [38]. We are confident of the ability of the phantom to estimate knee moments and stiffnesses provided by knee exoskeletons reliably.

Our results of assistive force transmission to the joint are exclusive to our exoskeleton, lower limb, and interface. Unlike our device, a passive-elastic knee exoskeleton designed to assist human walking would require a clutch to disengage the spring during leg swing [12]. The engagement and disengagement of the spring could cause impact forces not reflected in our assistance profile results. An exoskeleton assisting the knee in stance may also provide a higher stiffness to the joint. Our device provided a maximum knee moment comparable to the maximum biological knee moment in the stance phase of children over a larger range of motion (Fig. 7). Besides exoskeleton actuation, the interface also plays a role in transmitting forces to the musculoskeletal system. For example, a larger interface area will exert a smaller pressure and gel compression. Strategic placement of the braces near bony landmarks can reduce soft-tissue deformation but may be uncomfortable for the user. Besides surface area and position, the interface material also plays a role in force transmission. We used semi-rigid 3D-printed braces custom-made to the phantom shape. However, it can be time-consuming to 3D-scan, design, and 3D-print custom braces for every subject wearing the exoskeleton. Alternative interfaces such as hook and loop, or BOA^®^ [39] straps are adjustable to fit several lower limb circumferences but can deform under loads, potentially absorbing and dissipating work. Future studies must assess the effects of interface properties on the efficiency of load transmission to the joint. Lastly, subject body parameters such as body mass index can affect assistance force transmission. We constructed the phantom from a 3D child model having a $17\;kg/m^{2}$ body mass index, approximately the mean value of a 9-year-old male child [40]. It is reasonable to expect that a higher body mass index will lead to a higher energy dissipation at the exoskeleton interface due to soft-tissue deformation and vice versa.

Although the mechanical phantom provides valuable and reliable insight into exoskeleton assistance, it does not represent the lower-limb perfectly. For instance, the phantom knee is a monocentric revolute joint, whereas an actual human knee is polycentric with additional degrees of freedom outside the sagittal plane [41]. As a result, an exoskeleton worn by a subject may have misalignments not previously captured by the phantom. These uncaptured misalignments may lead to parasitic forces that result in shearing the leg’s soft tissues, user discomfort, and altered joint mechanics [42]. A monocentric hinge will have higher magnitude misalignments than a polycentric joint relative to a real human knee. A future iteration of the phantom may include a more physiologically accurate polycentric knee joint [43]. Nevertheless, our phantom provides a relatively accurate framework for initially testing knee exoskeleton prototypes before human subject testing. The ballistic gel is another point of simplification. Although ballistic gel provides a fair simulation of human soft-tissue compression, its frictional coefficient and viscoelasticity are physiologically different [32]. MyotonPro measurements revealed that our 20% (ballistic powder weight per water volume) ballistic gel had a ~380 N/m dynamic stiffness at 21 °C. For comparison, healthy children aged 5–7 years have a dynamic stiffness at the lower limb as tested by the MyotonPro in the range of 172.5 – 268.8 N/m depending on the muscle area probed [44]. However, researchers can easily change the gel stiffness of the phantom by using different gel concentrations (Fig. 10). Different clinical populations and age groups will have different soft-tissue stiffnesses and it is at the discretion of the researchers to choose the ballistic gel density that best captures the physiology of their research population [45], [46]. Additionally, ballistic gel is a relatively slippery material. Other mechanical phantoms use alternative materials such as polyurethane gels and polyether foam rubber to simulate soft-tissue deformation and interface friction [47], [48]. Because exoskeleton migration across the lower limb would affect the assistive movement, devices tested on the ballistic gel phantom should measure if there is migration and reduce it accordingly [49]. In the experiments here, we assumed that exoskeleton assistance does not affect the joint kinematics of walking. Human users might alter their kinematic movement pattern over time and practice. This could be simulated by the robotic controller in future studies. While our mechanical phantom can simulate knee kinematics, it cannot reproduce the joint kinetics caused by muscle contractions [50]. Unlike a human lower limb, the ballistic gel of the phantom has a constant stiffness throughout its surface and gait cycle, which will change its soft-tissue stiffness and potential energy stored relative to a human lower limb. Lastly, our phantom cannot provide other user-defined metrics such as device comfort, user satisfaction, and assistance perception. While not a perfect representation of a human lower limb, our phantom provides a more accurate testing model than most other mechanical knee simulators by incorporating soft-tissue deformation.

There are many advantages for testing an exoskeleton on a mechanical phantom. Being able to conduct medical device testing safely is crucial for product development, especially with vulnerable populations such as children. The phantom provides a risk-free environment for testing the mechanical integrity of the knee exoskeleton. The lack of human subject recruitment and testing can also speed up exoskeleton iteration. We can replicate most sagittal plane knee trajectories other than knee hyperextension due to a mechanical singularity at full knee extension. For example, we could use the phantom to test knee exoskeletons that assist children with crouch gait caused by cerebral palsy, the most common neuromuscular disorder in children [51], [52]. In addition to fast iteration, the phantom provides direct measurements of exoskeleton assistance that would otherwise be difficult to estimate from a subject as the total joint moment is a mixture of biological and exoskeleton components [19]. Furthermore, device assistance metrics obtained from the phantom can provide insights to engineers on how to improve the efficacy of assistive moment transfer to the joint. Active exoskeletons relying on external power sources could benefit from prolonged battery life by reducing the energy lost at the interface. Passive devices using elastic energy storage and return could use a lower stiffness spring to provide the same assistive moment, reducing device weight. Ultimately, our phantom should accelerate the design process, provide a reliable means to assess mechanical integrity, and ultimately improve the design of lower limb exoskeletons.

## Supplementary Material

supp2-3276424

supp1-3276424

## Figures and Tables

**TABLE I:** Inverse Dynamics Model Variables

Symbol	Definition	Unit

$l_{f}$	femur linkage length	m
$l_{t}$	tibia/fibula linkage length	m
$I_{f}$	femur linkage inertia	$kg\;m^{2}$
$I_{t}$	tibia/fibula linkage inertia	$kg\;m^{2}$
$m_{f}$	femur linkage mass	kg
$m_{t}$	tibia/fibula linkage mass	kg
g	gravitational acceleration	$m/s^{2}$
$p_{f}$	femur linkage center of mass to knee distance	m
$p_{t}$	tibia/fibula linkage center of mass to knee distance	m
$\theta_{f}$	femur linkage global angle	rad
$\theta_{t}$	tibia/fibula linkage global angle	rad
$\ddot{\theta_{f}}$	femur linkage angular acceleration	$rad/s^{2}$
${\ddot{\theta }}_{t}$	tibia/fibula linkage angular acceleration	$rad/s^{2}$
${\ddot{x}}_{f}$	femur linkage center of mass horizontal acceleration	N
${\ddot{y}}_{f}$	femur linkage center of mass vertical acceleration	N
${\ddot{x}}_{t}$	tibia/fibula linkage center of mass horizontal acceleration	N
${\ddot{y}}_{t}$	tibia/fibula linkage center of mass vertical acceleration	N
$F_{ax}$	horizontal ankle joint force	N
$F_{ay}$	vertical ankle joint force	N
$F_{kx}$	horizontal knee joint force	N
$F_{ky}$	vertical knee joint force	N
$F_{hx}$	horizontal hip joint force	N
$F_{hy}$	vertical hip joint force	N
M	knee moment	Nm

**TABLE II:** Phantom Limb Body Segment Parameters

Parameter	No spring/brace	Brace	Braceless

$I_{f}\left(kg\;m^{2}\right)$	1.1 × 10^−2^	1.3 × 10^−2^	1.3 × 10^−2^
$I_{t}\left(kg{\;m}^{2}\right)$	1.4 × 10^−2^	1.7 × 10^−2^	1.6 × 10^−2^
$w_{f}(N)$	13.1	16.2	15.4
$w_{t}(N)$	24.1	26.9	25.8
$p_{f}(m)$	0.13	0.11	0.11
$p_{t}(m)$	0.15	0.15	0.15

**TABLE III:** Phantom Tracking Error

Condition	Root Mean Squared Error

Brace/Target	3.6°
Braceless / Target	4.1°

**TABLE IV:** Assistance Profile Linear Regression Variables

Parameter	Slope (*Nm/rad*)	x-Intercept (°)	AUC

Brace	14.5 ± 0.01	6.77	0.24
Braceless	15.9 ± 0.02	2.44× 10^−2^	0.15

**TABLE V:** Adjusted Assistance Profile Linear Regression Variables

Parameter	Slope (*Nm/rad*)	x-Intercept (°)	AUC

Brace	15.2 ± 0.01	−0.884	0.23
Braceless	15.9 ± 0.02	2.44× 10^−2^	0.15
